# Health impact assessment of port-sourced air pollution in Barcelona

**DOI:** 10.1371/journal.pone.0305236

**Published:** 2024-08-30

**Authors:** Natalie Mueller, Marta Cirach, Albert Ambros, Carolyn Daher, Mark Nieuwenhuijsen, Xavier Basagaña

**Affiliations:** 1 ISGlobal, Barcelona, Spain; 2 Universitat Pompeu Fabra (UPF), Barcelona, Spain; 3 CIBER Epidemiología y Salud Pública (CIBERESP), Madrid, Spain; University of Coimbra: Universidade de Coimbra, PORTUGAL

## Abstract

**Introduction:**

Air pollution is a major health risk factor. Ports might be an understudied source of air pollution.

**Methods:**

We conducted a spatial health impact assessment (HIA) of port-sourced air pollution for Barcelona for 2017 at the neighbourhood level. Total NO_2_ and PM_10_ and port-sourced NO_2_, PM_10_ and PM_2.5_ concentrations were available through the ADMS-Urban model. Population data, mortality and morbidity data, and risk estimates were obtained. We followed standard HIA methodologies and calculated relative risks and impact fractions for 1.35 million adults living in 73 neighbourhoods.

**Results:**

The city-wide mean total NO_2_ and PM_10_ concentrations were 37.88 μg/m^3^ (range: 19.61–52.17 μg/m^3^) and 21.68 μg/m^3^ (range: 17.33–26.69 μg/m^3^), respectively, of which 7% (range: 2–36%) and 1% (range: 0–7%) were port-sourced, respectively. The mean port-sourced PM_2.5_ concentration was 0.19 μg/m^3^ (range: 0.06–1.38 μg/m^3^). We estimated that 1,123 (PI: 0–3,060) and 1,230 (95% CI: 0–2,566) premature deaths were attributable to total NO_2_ and PM_10_, respectively, of which 8.1% (91; PI: 0–264) and 1.1% (13; 95% CI 0–29) were attributable to port-sourced NO_2_ and PM_10_, respectively. 20 (95% CI: 15–26) premature deaths were attributable to port-sourced PM_2.5_. Additionally, a considerable morbidity burden and losses in life expectancy were attributable to port-sourced air pollution. Neighbourhoods closest to the port in the south-east were most adversely affected, gradually decreasing towards the north-west.

**Conclusions:**

The port is an understudied air pollution source in Barcelona with strong health impacts. Cities need local insight into health risk factors, their sources, attributable burdens and distributions for defining targeted policies.

## Introduction

The increasing awareness and concern about climate change have led to great attention in science and policy to the links between environment and health. Air pollution is well established as a main global environmental health problem. Due to innovation and economic growth, since the 1960s international trade has grown rapidly and nowadays goods are available from all over the world [1]. Sea transport accounts for 80% of goods transported worldwide, moving 10 billion tonnes of cargo annually [2]. Sea transport allows for much larger volumes to be transported than alternative road, rail or air transport, and recent estimates foresee a demand growth of almost 40% for sea transport by 2050 [3]. While sea transport allows rapid mass movement of goods, it comes at high costs of sea and air pollution.

At the EU level, sea transport accounts for about 3–4% of the EU’s total CO_2_ emissions [4] and also emits other air pollutants, such as particles (PM), nitrogen oxides (NO_x_) and sulphur oxides (SO_x_), known to be harmful to human health. Air pollution epidemiology has a long tradition and the evidence base for air pollution being linked to mortality, cardiovascular disease (CVD), cerebrovascular and metabolic diseases, is well established [5–9].

A vast amount of research focused on traffic-related air pollution (TRAP) and the associated health burden, but less is known about shipping and port-sourced air pollution in cities. Recent research evidence indicates that worldwide up to 265,000 premature deaths annually, i.e., 0.5% of global mortality, could be due to global shipping [10]. For over 850 European cities, it was estimated that 5.5% of PM_2.5_ related mortality (10,116 deaths) and 9.7% of NO_2_ related mortality (9,020 deaths) could be due to shipping [11]. This evidence, together with a recent scoping review on the health impacts of shipping and port-sourced air pollution, positions sea transport as an important source of air pollution causing a considerable health burden [12]. Also, port activities alone, like manoeuvring cargo and needed machinery, and trucks and trains, are believed to considerably contribute to port-sourced air pollution level [13].

While global and large-scale studies are important to demonstrate the overall health impact and burden a risk factor might cause, city-level studies are needed to gain better insight into the precise risk and distribution a factor might present for local populations. Local evidence is needed for policy prioritisation and formulation of targeted intervention strategies, which city councils are more agile to do than national governments.

Using the case of air pollution in Barcelona, Spain, road transport, especially in the densely-trafficked city centre has been identified as an important source of pollution [14, 15]. However, also other sources, such as regional concentrations, industry, construction, domestic sources, as well as shipping and port activities, are believed to make important contributions to overall air pollution levels in the city [16, 17]. With international trade, economies and societies recovering from the Covid-19 blow, today, the port of Barcelona is a major logistics hub of Spain and in 2022 moved over 70 million tonnes of goods during almost 9,000 ship calls [18]. Moreover, 3.9 million passengers passed through the port, of which 2.3 million passengers visited Barcelona during 800 cruise ship calls [18]. The vast amount of ship calls, tonnes of goods transported and passengers passing through make the port of Barcelona an important, potentially understudied, source of air pollution for the city. While some evidence already exists for the port of Barcelona contributing to total city-level air pollution levels [19, 20], none of these studies studied the spatial distribution and associated health burden within the city, which is important for pinpointing hotspots of environmental and health burdens. Therefore, the objectives of this study were, first, to estimate spatially the contribution of the Barcelona port, including shipping and port activities, to local air pollution concentrations, and secondly, to estimate spatially the attributable health burden in terms of mortality and morbidity impacts.

## Methods

This study was set in Barcelona, located at the north-eastern Iberian Peninsula of the Mediterranean coast. It was decided to conduct the study for 2017, as data was available and for more recent years (e.g., 2020–2023) it could not be ruled-out to still reflect Covid-19 pandemic effects and interruptions of international trade and shipping.

First, a brief description situating the port of Barcelona in the context of the city is provided. Second, we explain how ambient air pollution concentrations from all sources, and secondly, port-sourced concentrations were estimated, using the ADMS-Urban model provided by Barcelona Regional (BR). BR is the Metropolitan Agency for Urban Development and Infrastructures and forms a consortium with the Barcelona City Council and other public stakeholders, such as the Port of Barcelona, and assists in undertaking strategic urban development analyses. Third, we describe the applied health impact assessment (HIA) framework, following the comparative risk assessment framework (CRA) [21], to estimate the port-sourced attributable mortality and morbidity burdens.

### The port of Barcelona in the city context

The port of Barcelona is located on the south-east end of the city, placed at the foot of the Montjuïc mountain and stretches the coast between “la Barceloneta” neighbourhood in the north and the Llobregat river in the south. Adjacent neighbourhoods from north to south are “la Barceloneta”, “el Gòtic”, “l’Antiga Esquerra de l’Eixample”, “el Poble Sec” and “la Marina del Prat Vermell” (S1 Fig), all neighbourhoods composed of socioeconomically more deprived populations [22]. In terms of technical characteristics, the port has three business units that occupy different spaces: the commercial port, the logistics port and the Port Vell, or Old Port, for nautical and sport uses [23]. In terms of land area, the port occupies 1,112 ha and its wharves and berths have a length of over 23 km [23]. There are 3 container and multipurpose terminals, 2 car terminals, 1 coffee and cocoa terminal, 9 liquid bulk terminals, 5 dry bulk terminals, 3 ferry terminals and 7 cruise terminals [23]. As described above, the port of Barcelona is an important port in the Mediterranean and in 2022, over 70 million tonnes of goods and almost 4 million passengers passed through the port, during almost 9,000 ship calls. The port represents an important source of atmospheric pollution to the city that is often transported across the city by the sea breeze during the day. Due to Barcelona’s topography (sea to the east, Collserola mountains to the west), the transport and dispersion of pollutants within the city is largely controlled by fluctuating coastal winds, which blow from the sea land inwards during the day (diurnal breeze) and lighter winds from the land to the sea at night (night breeze) [24, 25].

### Air pollution from all sources and port-sourced concentrations

Using the ADMS-Urban model, air pollution from all sources (NO_2_, PM_10_) and port-sourced (NO_2_, PM_10_, PM_2.5_) concentrations, including shipping and port activities, were estimated for 2017. The ADMS-Urban model is a dispersion model developed by Cambridge Environmental Research Consultants (CERC) to work on street level in urban and metropolitan environments. It is developed based on an advanced Gaussian model for the modelling of pollution concentrations and represents the full range of source types occurring in urban areas and considers important determinants such as complex urban morphologies, meteorological data, such as wind speed, wind direction or temperature, but also traffic flows and background ambient concentrations [26].

To study Barcelona port-sourced air pollution, the inventory emission sources provided by the Port of Barcelona and processed by BR were ships (traffic and auxiliary), auxiliary land machinery and road transport [27]. To study city air pollution concentrations from all sources, the inventory of emissions and modelling was provided by the Barcelona City Council under the Plan for Improvement of Air Quality in Barcelona 2015–2018 (Pla de millora de la qualitat de l’aire Barcelona (PMQA) 2015–2018) and included, in addition to emissions from the port, emissions from road transport, the industrial, commercial and domestic sectors, the airport, and fugitive and regional background emissions [28].

For this study, mean annual air pollution concentrations were aggregated at neighbourhood level (n = 73).

### Health impact assessment (HIA)

To assess the health burden attributable to total and port-sourced air pollution concentrations for Barcelona in 2017, we conducted a spatial HIA at the neighbourhood level (n = 73) (S1 Fig), following the CRA, a standard HIA methodology [21]. We considered the Barcelona 2017 adult population aged 20 years and older and obtained population data from the city council (S1 Table) [29]. We aggregated the Barcelona adult population aged 20 years and older data for 2017 in 5-year age bounds for the 73 neighbourhoods. The choice of included morbidity outcomes was based on meta-analysed exposure-response functions (ERFs) being available, so that generalizability and extrapolation of risk estimates could be assumed, representing also the breadth of health outcomes that air pollution is associated with, emphasizing the urgency to reduce air pollution levels.

Following the CRA approach, a) we defined the exposure measures (NO_2_, PM_10_, PM_2.5_); b) we identified baseline incidence statistics for health outcomes of interest for which a strong evidence base exists (i.e., natural-cause mortality, CVD, hypertension, type 2 diabetes and stroke) [30–32] (Table 1).

**Table 1:** Incidence rates for selected health outcomes.

Health outcome	Sex	Location	Age	Year	Incidence/ 100,000 population	Expected cases	Reference
Natural-cause mortality	Both	Barcelona	≥20	2016	1,093	14,751	[30]
CVD (ICD-10 codes: I20-I25)	M	Barcelona	≥35	2015	642	3,057	[31]
	F	Barcelona	≥35	2015	278	1,572	
Hypertension (ICD-10 codes: I10-I15)	M	Barcelona	≥35	2015	1,595	7,595	[31]
	F	Barcelona	≥35	2015	1,602	9,059	
Type 2 diabetes (ICD-10 code: E11)	M	Barcelona	≥35	2015	774	3,686	[31]
	F	Barcelona	≥35	2015	560	3,167	
Stroke	M	Spain	≥65	2010	690	961	[32]
	F	Spain	≥65	2010	370	775	

CVD = cardiovascular disease; ICD-10 = international classification of disease, version 2010; F = female; M = male

The baseline incidence statistics for selected health outcomes were accessed in May 2019 through local health registries (i.e. Agència de Salut Pública de Barcelona (ASPB) and Sistema d’Informació per al Desenvolupament de la Investigació en Atenció Primària (SIDIAP) [30, 32]). (The provided health incidence statistic is de-identified and no identification of individuals is possible); c) we selected meta-analysed ERFs from the literature that quantify the strength of association between the air pollution concentrations (NO_2_, PM_10_, PM_2.5_) and the development of the health outcomes (S2-S4 Tables); d) we combined exposure data (total and port-sourced air pollution) with the population at the Barcelona neighbourhood level (n = 73), the ERFs and incidence statistics to quantify the attributable proportional health burden. As exclusively secondary population and health data were used from local registries and repositories, no ethical approval for this study was required.

### Life table analysis

We estimated average changes in life expectancy attributable to total and port-sourced air pollution, using life tables for Catalonia (2013–2017) [33] applying life table methods, as first described by Brunekreef (1997) for air pollution [34]. We estimated average changes in life expectancy for the Barcelona population ≥ 20 years by changing the probabilities of dying because of city-wide mean exposure to total and port-sourced air pollution (S5 Table). The life table analysis represents the expected average change in life expectancy one could expect if reported total and port-sourced air pollution concentrations were set equal to zero in comparison to baseline (i.e. avoided loss in life expectancy).

## Results

As of 2017, Barcelona had a population of 1.35 million adult residents aged 20 years and older (S1 Table), living in the 73 administrative neighbourhoods of the city (S1 Fig).

### Total and port-sourced air pollution

**Fig 1:**
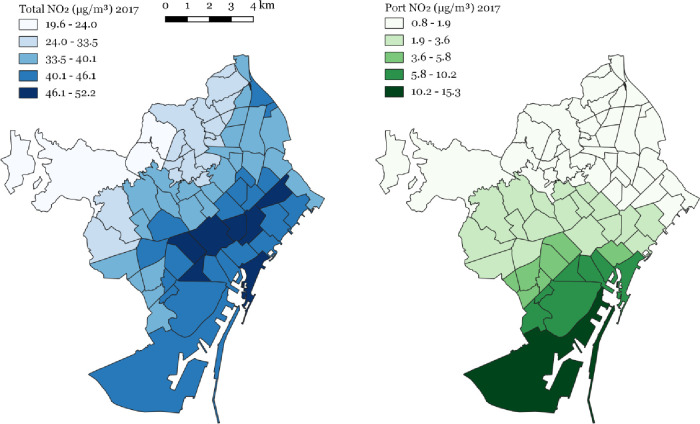
Total and port-sourced NO_2_ (μg/m^3^) concentrations 2017 at the Barcelona neighbourhood level.

In 2017, city-wide mean total NO_2_ concentrations were 37.88 μg/m^3^, ranging between 19.61 μg/m^3^ and 52.17 μg/m^3^ (Table 2 and Fig 1).

**Table 2:** City-wide total NO_2_, PM_10_ and port-sourced NO_2_, PM_10_ and PM_2.5_ concentrations, 2017.

Pollutant	Total (μg/m^3^)	Port (μg/m^3^)	Contribution port to total (%)
Annual mean NO_2_ (range)	37.88 (19.61–52.17)	2.81 (0.76–15.29)	7.08 (1.97–36.22)
Annual mean PM_10_ (range)	21.68 (17.33–26.69)	0.22 (0.07–1.59)	0.99 (0.31–7.17)
Annual mean PM_2.5_ (range)	-	0.19 (0.06–1.38)	-

**Fig 2:**
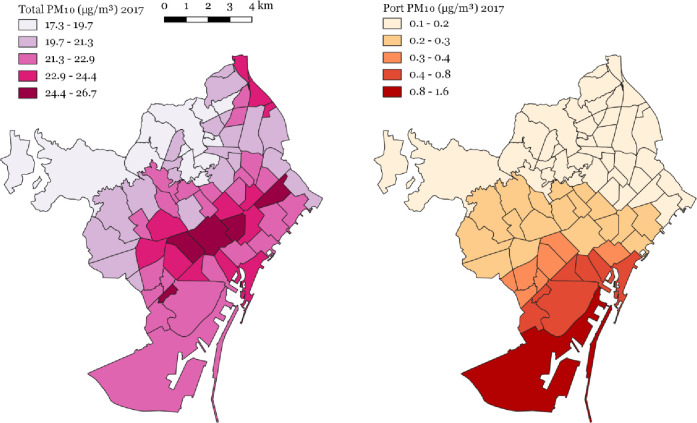
Total and port-sourced PM_10_ (μg/m^3^) concentrations 2017 at the Barcelona neighbourhood level.

**Fig 3:**
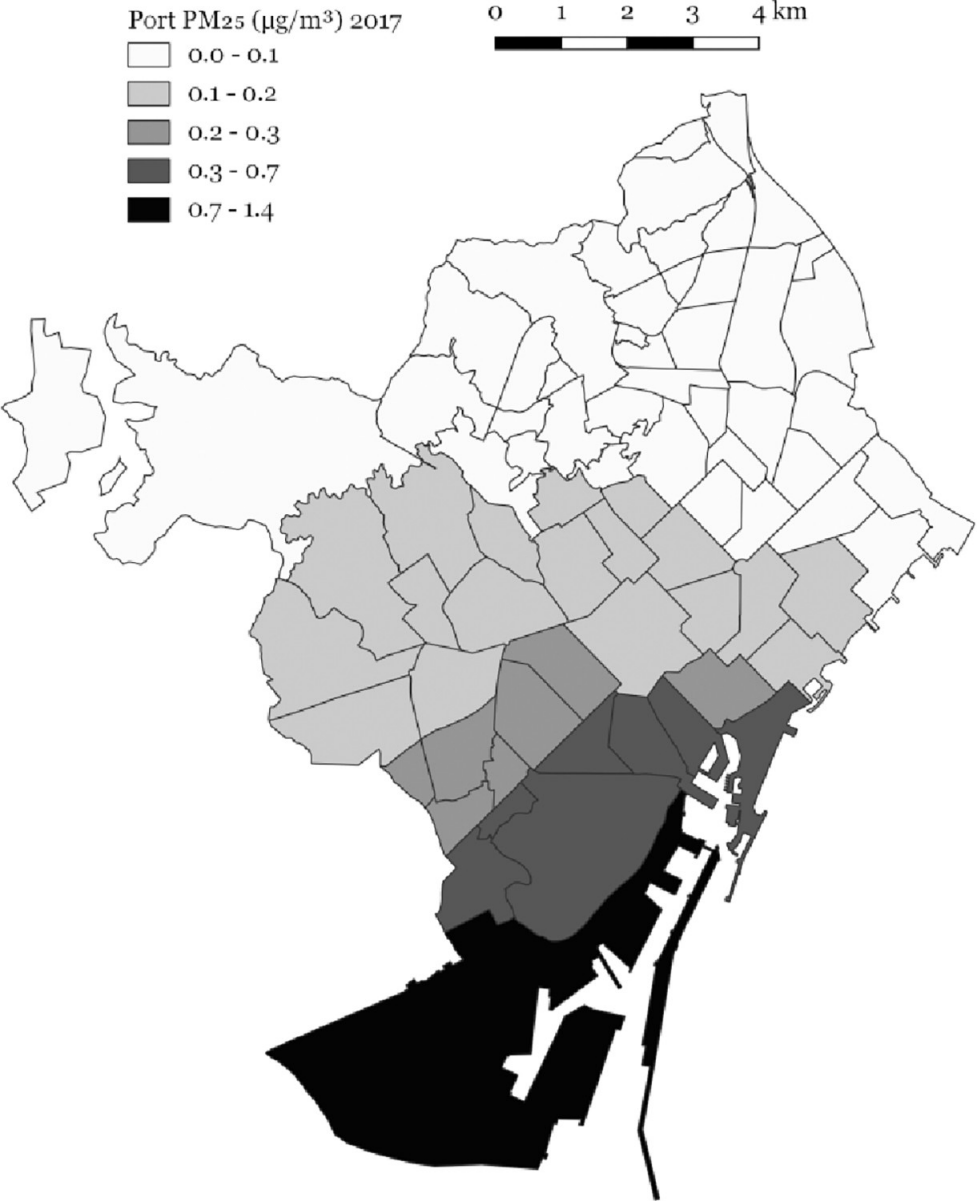
Port-sourced PM_2.5_ (μg/m^3^) concentrations 2017 at the Barcelona neighbourhood level.

City-wide mean port NO_2_ concentrations were 2.81 μg/m^3^, ranging between 0.76 μg/m^3^ and 15.29 μg/m^3^. City-wide mean PM_10_ total concentrations were 21.68 μg/m^3^, ranging between 17.33 μg/m^3^ and 26.69 μg/m^3^ (Table 2 and Fig 2). City-wide mean port PM_10_ concentrations were 0.22 μg/m^3^, ranging between 0.07 μg/m^3^ and 1.59 μg/m^3^. City-wide mean port PM_2.5_ concentrations were 0.19 μg/m^3^, ranging between 0.06 μg/m^3^ and 1.38 μg/m^3^ (Table 2 and Fig 3).

In 2017, the port contributed 7% of total NO_2_ concentrations, ranging between 2% and 36%, and 1% of total PM_10_ concentrations, ranging between 0% and 7%.

### Estimated health impacts

We estimated that in 2017, 1,123 (PI: 0–3,060) premature deaths were attributable to total NO_2_ concentrations in the city of Barcelona, of which 91 deaths (PI: 0–264) (7 deaths/ 100,000 persons) or 8.1% were attributable to port-sourced NO_2_ concentrations (Table 3).

**Table 3:** Estimated attributable cases related to total and port-sourced NO_2_, PM_10_ and PM_2.5_.

Health outcome	Attributable cases				
	Total NO_2_	Port NO_2_	Total PM_10_	Port PM_10_	Port PM_2.5_
Natural-cause mortality (PI)	1,123 (0–3,060)	91 (0–264)	1,230 (0–2,566)	13 (0–29)	20 (14–26)
CVD M (95% CI)	131 (72–188)	10 (6–15)	678 (67–1,190)	8 (1–16)	15 (0–32)
CVD F (95% CI)	67 (37–96)	5 (3–7)	349 (34–612)	4 (0–8)	7 (0–16)
Hypertension M (95% CI)	947 (150–1,639)	78 (12–141)	835 (572–1,084)	9 (6–12)	-
Hypertension F (95% CI)	1,128 (178–1,952)	90 (14–164)	997 (683–1,293)	11 (7–14)	-
Type 2 diabetes M (95% CI)	971 (0–1,705)	86 (0–172)	-	-	7 (1–12)
Type 2 diabetes F (95% CI)	833 (0–1,463)	72 (0–144)	-	-	6 (1–10)
Stroke M (95% CI)	138 (0–475)	11 (0–47)	-	-	6 (0–17)
Stroke F (95% CI)	112 (0–384)	9 (0–38)	-	-	5 (0–14)

CVD = cardiovascular disease; F = female; M = male; PI = prediction interval; 95% CI = 95% confidence interval

Those 91 port-sourced NO_2_ attributable deaths accounted for 0.62% of total natural-cause mortality expected in the city. We estimated that 131 (95% CI: 72–188) cases of CVD in males and 67 (95% CI: 37–96) cases in females were attributable to total NO_2_ concentrations, of which 10 (95% CI: 6–15) cases in males and 5 (95% CI: 3–7) cases in females were attributable to port-sourced NO_2_ concentrations. We estimated that 947 (95% CI: 150–1,639) cases of hypertension in males and 1,128 (95% CI: 178–1,952) cases in females were attributable to total NO_2_ concentrations, of which 78 (95% CI: 12–141) cases in males and 90 (95% CI: 14–164) cases in females were attributable to port-sourced NO_2_ concentrations. We estimated that 971 (95% CI: 0–1,705) cases of type 2 diabetes in males and 833 (95% CI: 0–1,463) cases in females were attributable to total NO_2_ concentrations, of which 86 (95% CI: 0–172) cases in males and 72 (95% CI: 0–144) cases in females were attributable to port-sourced NO_2_ concentrations. We estimated that 138 (95% CI: 0–475) cases of stroke in males and 112 (95% CI: 0–384) cases in females were attributable to total NO_2_ concentrations, of which 11 (95% CI: 0–47) cases in males and 9 (95% CI: 0–38) cases in females were attributable to port-sourced NO_2_ concentrations.

We estimated that 1,230 (95% CI: 0–2,566) premature deaths were attributable to total PM_10_ concentrations in the city of Barcelona, of which 13 deaths (95% CI: 0–29) (1 death/ 100,000 persons) or 1.1% were attributable to port-sourced PM_10_ concentrations (Table 3). Those 13 port-sourced PM_10_ attributable deaths accounted for 0.09% of total natural-cause mortality expected in the city. We estimated that 678 (95% CI: 67–1,190) cases of CVD in males and 349 (95% CI: 34–612) cases in females were attributable to total PM_10_ concentrations, of which 8 (95% CI: 1–16) cases in males and 4 (95% CI: 0–8) cases in females were attributable to port-sourced PM_10_ concentrations. We estimated that 835 (95% CI: 572–1,084) cases of hypertension in males and 997 (95% CI: 683–1,293) cases in females were attributable to total PM_10_ concentrations, of which 9 (95% CI: 6–12) cases in males and 11 (95% CI: 7–14) cases in females were attributable to port-sourced PM_10_ concentrations.

We estimated that 20 (95% CI: 14–26) premature deaths (2 deaths/ 100,000 persons) were attributable to port-sourced PM_2.5_ concentrations (Table 3). Those 20 port-sourced PM_2.5_ attributable deaths accounted for 0.14% of total natural-cause mortality expected in the city. We estimated that 15 (95% CI: 0–32) cases of CVD in males and 7 (95% CI: 0–16) cases in females were attributable to port-sourced PM_2.5_ concentrations. We estimated that 7 (95% CI: 1–12) cases of type 2 diabetes in males and 6 (95% CI: 1–10) cases in females were attributable to port-sourced PM_2.5_ concentrations. We estimated that 6 (95% CI: 0–17) cases of stroke in males and 5 (95% CI: 0–14) cases in females were attributable to port-sourced PM_2.5_ concentrations.

**Fig 4:**
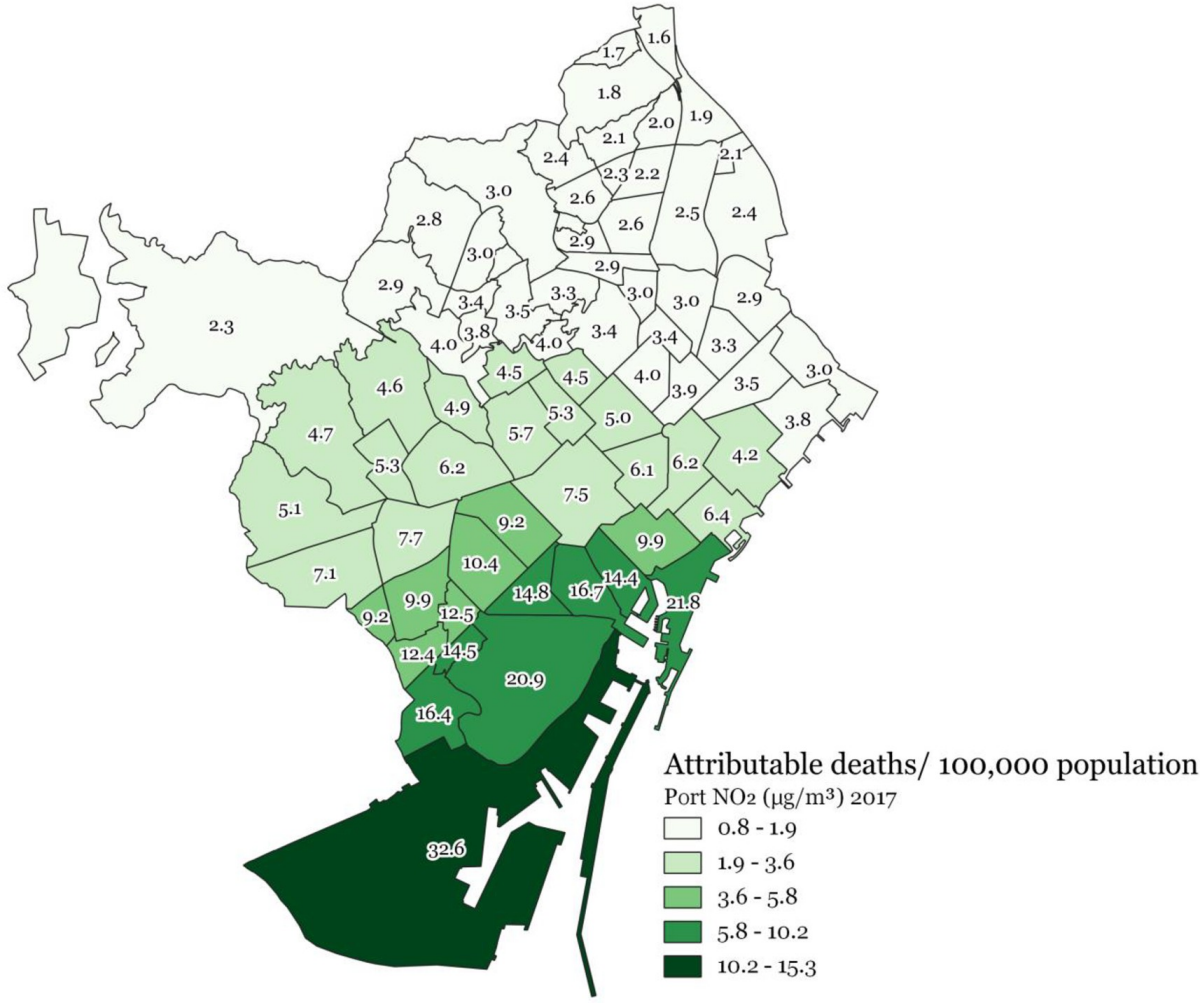
Port-sourced NO_2_ (μg/m^3^) attributable deaths/100,000 population at the 2017 Barcelona neighbourhood level.

**Fig 5:**
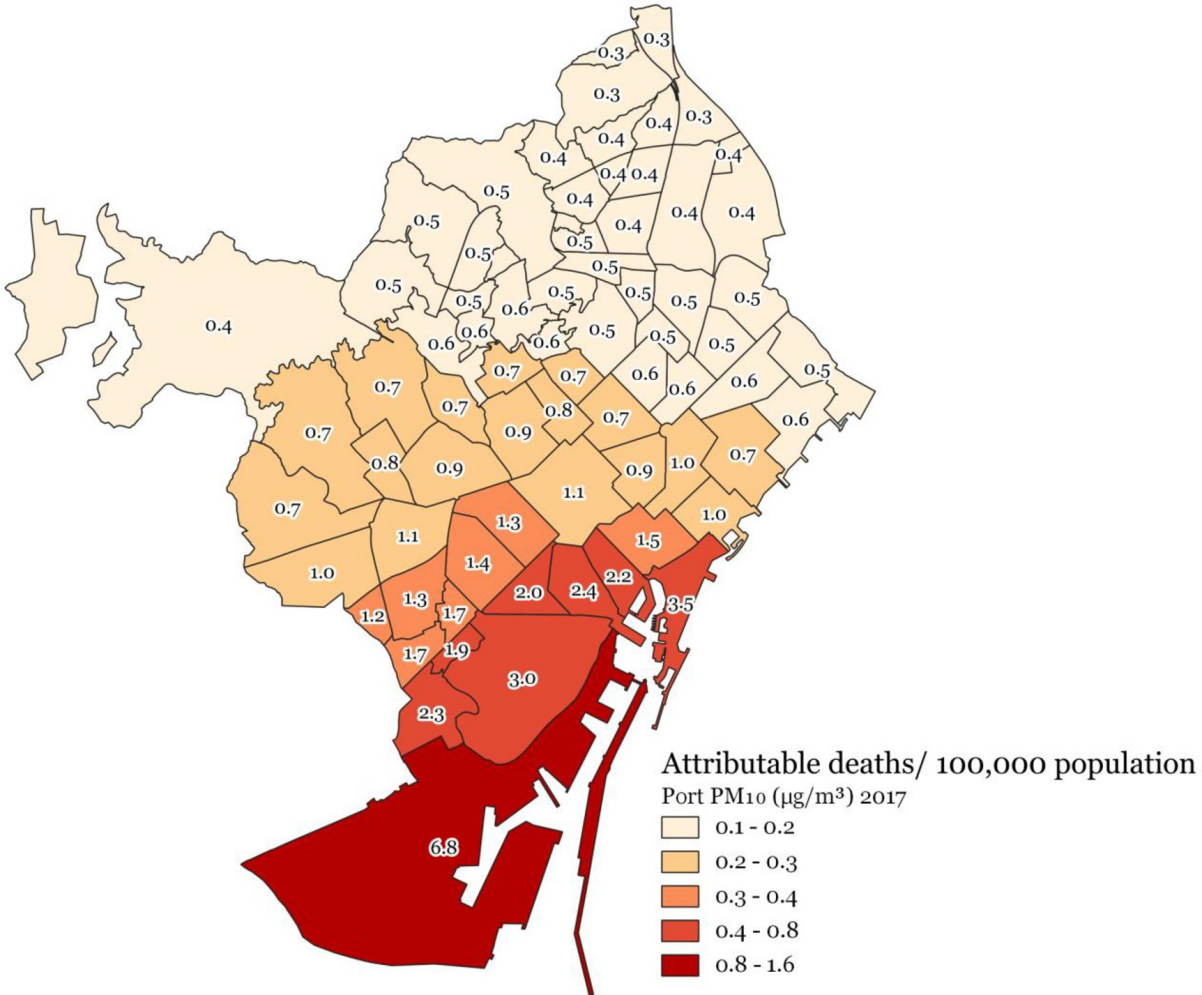
Port-sourced PM_10_ (μg/m^3^) attributable deaths/100,000 population at the 2017 Barcelona neighbourhood level.

**Fig 6:**
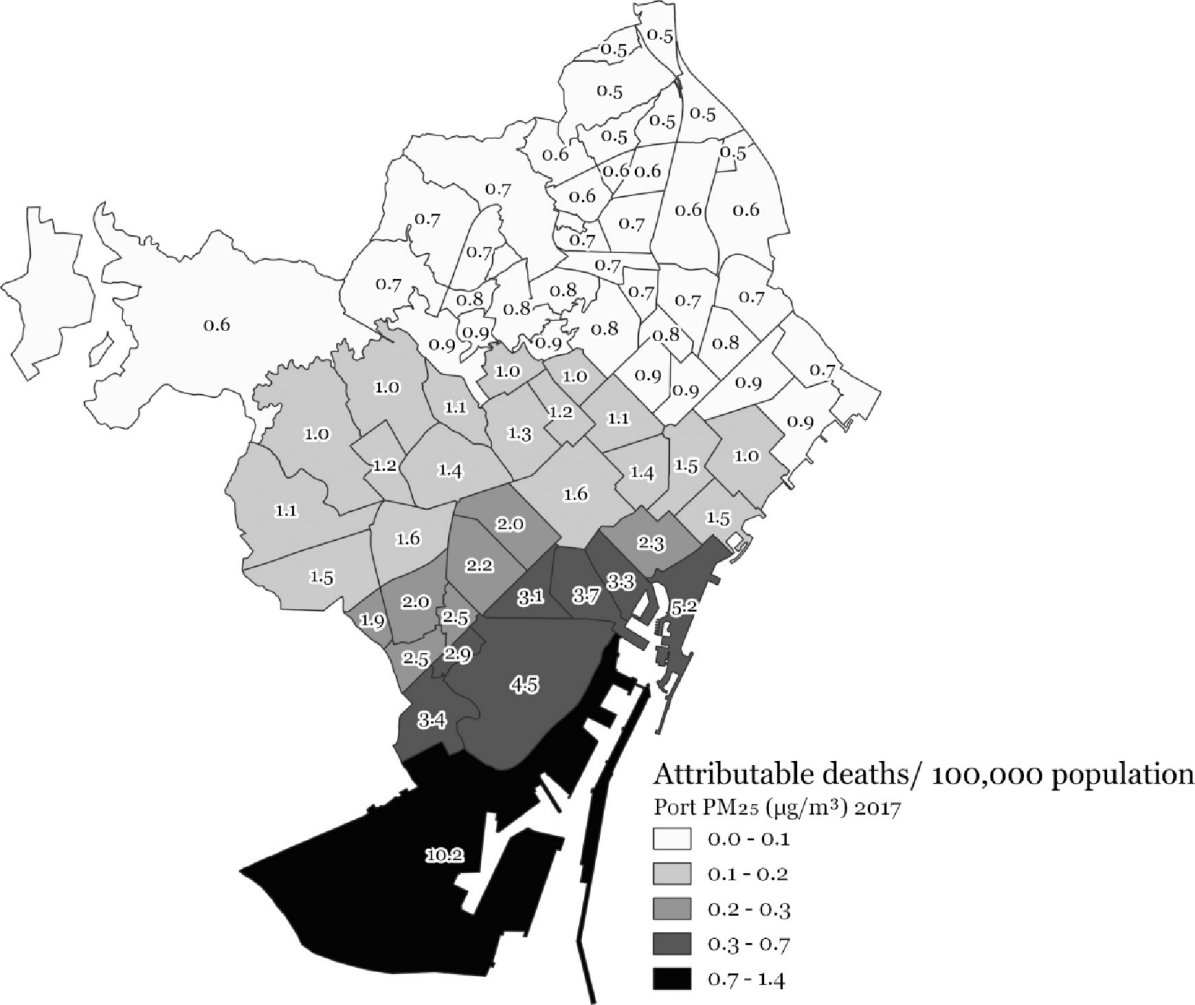
Port-sourced PM_2.5_ (μg/m^3^) attributable deaths/100,000 population at the 2017 Barcelona neighbourhood level.

The highest attributable health burdens (i.e., standardized attributable mortality rates) due to port-sourced air pollution were consistently found for all three pollutants for the neighbourhoods closest to the port, meaning the highest rates for neighbourhoods in the south-east of the city and then gradually decreasing for the neighbourhoods towards the north-west (Figs 4–6).

### Life tables

We estimated that Barcelona residents aged 20 years and older on average lose 271 days of their life expectancy because of exposure to city-wide mean NO_2_ levels (i.e. 37.88 μg/m^3^), of which 19 days are lost on average because of city-wide mean port-sourced NO_2_ exposure (i.e. 2.81 μg/m^3^) (S5 Table). Barcelona residents aged 20 years and older lose on average 310 days of their life expectancy because of exposure to city-wide mean PM_10_ levels (i.e. 21.68 μg/m^3^), of which 3 days are lost on average because of city-wide mean port-sourced PM_10_ exposure (i.e. 0.22 μg/m^3^). Barcelona residents aged 20 years and older lose on average 4 days of their life expectancy because of city-wide mean port-sourced PM_2.5_ exposure (i.e. 0.19 μg/m^3^).

## Discussion

This study provides a first insight into the health burden and distribution thereof associated with port-sourced air pollution in Barcelona, including shipping and other port activities. We estimated that 8.1% of the total mortality burden related to NO_2_ concentrations or 7 deaths/ 100,000 persons, 1.1% of the total mortality burden related to PM_10_ concentrations or 1 death/100,000 persons, and 2 deaths/100,000 persons related to PM_2.5_ concentrations can be attributed to shipping and port activities in Barcelona in 2017. In addition, a considerable morbidity burden and losses of life expectancy were associated with air pollution concentrations originating from the Barcelona port. The highest port-sourced air pollution concentrations and attributable health burdens for all three pollutants were found for neighbourhoods closest to the port (composed of socioeconomically more deprived populations), meaning highest standardized (mortality) rates for neighbourhoods in the south-east, gradually decreasing towards the north-west of the city (Figs 4–6).

The mortality impact estimate of our study of 8.1% of NO_2_ related mortality being port-sourced is in line with a large European city study of almost 900 cities that estimated 9.7% of NO_2_ related mortality to be due to shipping (and ports) [11]. Our study provides local evidence for the health burden, and the distribution thereof, associated with shipping and port activities in Barcelona, an understudied air pollution source with a considerable health impact. A previous study by Viana et al. 2020 estimated for Barcelona for 2011 at city-level that 1 μg/m^3^ of total PM_2.5_ concentrations were shipping-sourced, resulting in 60–177 attributable premature deaths [19], which is considerably higher than the mean 0.19 μg/m^3^ (range: 0.06–1.38 μg/m^3^) PM_2.5_ we found to be port-sourced for 2017, resulting in 20 attributable premature deaths. Likewise, our relative estimate of 2 deaths/ 100,000 persons being attributable to port-sourced PM_2.5_ is considerably lower than the relative shipping-sourced PM_2.5_ deaths estimated by Viana et al. 2020 and also Nunes et al. 2021, ranging between 4–13 deaths/100,000 persons [19, 20]. Both latter studies, however, used different emission inventories, methods and study years. Viana et al. 2020 estimates for 2011 were based on a receptor model that can provide source apportionment for PM_2.5_ [19] and Nunes et al. 2021 estimates for 2015 were based on a chemical transport model for the Iberian Peninsula [20], which unlike the ADMS-Urban dispersion model used in our study, does not provide spatially-distributed concentrations, which is important in HIA studies, as impacts are exposure, population and health status distribution dependent.

Until now, research emphasis has been put on TRAP in cities, which is the greatest source of air pollution (and noise) in Barcelona, with a large attributable health burden [11, 15, 35]. However, also other sources, such as shipping and port activities, need research and policy attention to minimize attributable environmental, climate and health burdens [11].

Cities, which carry local responsibility for the well-being of their residents and are more agile to act than national governments, need local evidence on the particular problems in their cities in order to provide targeted intervention strategies. In the context of foreseen increases in global trade and seaborne shipping demands [3], and hence linked increases in air pollution, regulating shipping and port emissions to reduce adverse environmental and health impacts becomes a matter of policy urgency. While generally progress has been made in reducing air pollution from the transport sector, largely due to policies addressing road transport, shipping and aviation are two subsectors for which emissions have actually increased. For Europe, shipping-sourced CO, NO_x_, SO_x_ and PM levels were all between 12–30% higher in 2019 compared to 1990 [36].

Coastal populations, especially European coastal populations, which includes Barcelona residents, were previously found to receive the highest shipping-sourced air pollution levels, and thus manifest the highest attributable health burdens [12]. In Europe, coastal regions are densely populated and heavy-trafficked shipping routes run in close proximity to shore [12, 37, 38]. As opposed to Asia, where shipping routes are located further away from shore, in the Mediterranean Sea, shipping routes are located in direct coastal proximity [37]. Moreover, the Mediterranean Sea carries a double-burden of being highly cargo and cruise ship trafficked, with estimations indicating that 24% of the global ship fleet and 17% of the cruise ship fleet ply the Mediterranean Sea [39]. The Mediterranean Sea connects Europe with Asia and thus covers a major trading route, while at the same time, the Mediterranean is the second largest market for the cruise ship industry after the Caribbean [40], as seen by the 800 cruise ship calls that Barcelona receives every year.

### Policy implications

The International Maritime Organisation (IMO) is the United Nations (UN) specialized agency responsible for the safety and security of shipping and the prevention of associated atmospheric and maritime pollution. UN member states are responsible for their own shipping industries, but are obliged to comply with IMO regulations. The International Convention for the Prevention of Pollution from Ships (MARPOL) by the IMO, first entered into force in 2005, provides in Annex VI the regulations for reducing shipping sourced emissions [41]. Since 2013, new ships must comply with the Energy Efficiency Design Index (EEDI), aimed at improving ship engines and equipment. Also, all ships must comply with the Ship Energy Efficiency Management Plan (SEEMP) that defines mechanisms to improve the energy efficiency of ships [42]. Moreover, under MARPOL Annex VI, the IMO has established emission control areas (ECAs) for sulphur oxides (SO_x_) (i.e. SECA) and nitrogen oxides (NO_x_) (i.e. NECA), forcing shipping companies to lower SO_x_ and NO_x_ emissions, respectively, and switching to cleaner (more expensive) fuels. Also, in 2020, IMO introduced an important sulphur fuel content cap and globally requires the sulphur fuel content to not exceed 0.1% in established SECAs and 0.5% in non-SECA waters [43]. Until now, there are four ECAs implemented by the IMO, the North American ECA, the US Caribbean ECA, the North Sea ECA and the Baltic Sea ECA [44]. The Mediterranean Sea, despite heavily ship-trafficked and adjacent to dense population centres, including Barcelona, is not an ECA yet, however, is adopted by IMO as of December 2022 to become an ECA for SO_x_ and PMs (i.e., SECA) by May 2025 [39], which is expected to have significant impacts for SO_x_ and PM reductions [10], but might not do much in terms of NO_x_ reductions [10, 20]. Therefore, continued advocacy for making the Mediterranean Sea also a NO_x_ ECA (i.e. NECA) in the near future is desirable.

Furthermore, there are European level regulations. The EU has adopted directives for lowering the sulphur fuel content to 0.1% for ships at berth for more than 2 h in all Union ports (Directive (EU) 2016/802). Moreover, as of 2025, the EU requires liquified natural gas (LNG), a distillate fuel, refuelling points and provision of shoreside electricity in all major Union ports (Directive, 2014/94/EU) [3], including Barcelona. These measures are meant to decrease shipping and port-sourced emissions and air pollution levels considerably.

Despite these efforts, compliance for switching to cleaner fuels for ships at berth and in ports is uncertain, as the costs can be high and penalties are low [45]. Since Mediterranean Sea is not a SECA yet, there is currently a risk of shipowners assigning older, more polluting ships to the Mediterranean Sea, resulting in high emissions and air pollution levels and attributable health burdens. Also, the 2020 IMO requirement for ships to lower their sulphur fuel content brings along certain challenges: the increased demand for cleaner fuels, also aggravated by the current energy crisis, leads to prices for cleaner fuels to increase [12]. This in return might lead shipowners to install scrubbers instead that can reduce air pollution levels to equivalent sulphur content levels, but heavily pollute the seawater. Also, improving the systems for monitoring ship design and operational performance, including EEDI and SEEMP and types of fuels, are needed and can contribute to more complete and standardized ship emission records that can help inform compliance and policy across different regions [46].

Local city-level policies with more restrictive regulations for ships at berth and port activities are needed to reduce emissions, air (and sea) pollution and attributable health burdens, which the port of Barcelona partially commits to, such as ambitious electrification and use of cleaner fuels (i.e., LNG and hydrogen) for ships and port activities and also tax benefits for cleaner ships [47]. In the lights of environmental justice and health equity implications, the need for local city-level policies is particularly important in the context of the Barcelona neighbourhoods closest to the port, which were found to receive the highest port-sourced air pollution concentrations and suffer the highest attributable health burdens, are neighbourhoods home to socioeconomically more deprived populations [22]. These socioeconomically more deprived populations already predispose a wide range of other risk factors (e.g. economic hardship, poor diets, etc.) that make them particularly vulnerable to environmental risk factors (i.e. air pollution) and associated health burdens. Thus, ambitious city-level policies to reduce port-sourced air pollution in Barcelona (e.g. reducing the number of cruise ship calls as has been proposed in the past [48]) would have a particularly important health-promoting impact for these disadvantaged populations.

### Methodological considerations

There are some methodological considerations that need to be acknowledged and are found in other similar HIA studies. All presented health impacts are estimates and need caution in interpretation, despite being based on best available epidemiological evidence: The estimated attributable health impacts of the different pollutants (i.e., NO_2_, PM_10_, PM_2.5_) should not be added together, as there is probably overlap of the pollutants causing health damage and it remains uncertain which one (or a combination of the pollutants) is the putative agent. In fact, the estimated heath impacts are probably an overestimation because each pollutant stands for the other pollutants, to some extent. To properly disentangle the risks attributable to the different pollutants, risk estimates from multi-pollutant models would be needed. Also, the estimated health impacts attributable to total air pollution concentrations of NO_2_ and PM_10_ need special caution in interpretation as the presented impact reflect the total attributable burden in comparison to zero air pollution. Zero air pollution, however, is unrealistic as natural sources of air pollution will always exist. This scenario was included to highlight that anthropogenic sources of air pollution, including shipping and port-sourced air pollution, need to be reduced as much as possible, because health effects of air pollution occur under established health thresholds [49].

Moreover, the Barcelona port-sourced air pollution was modelled considering air pollution coming from combustion sources (i.e., ships, auxiliary land machinery and road traffic), not considering e.g. secondary aerosols and other potential emissions. Also, other shipping and port-sourced pollutants with established health links such as SO_2_ or black carbon were not modelled. Therefore, the true contribution of the port to air pollution levels in the city is likely larger. In order to have a better idea of the real impact of port-sourced air pollution in Barcelona and complementing the modelled data, we recommend installing measurement stations in the south-eastern neighbourhoods closely situated to the port and measuring in real-time air pollution concentrations over time more accurately. In addition, source apportionment assessments are needed to better understand the chemical composition of PM, which is important because different toxic compounds of PM were shown to have varying health effects [50, 51]. We restricted our morbidity outcome choice to those outcomes for which high-quality ERFs for air pollution associations were available, while air pollution is also linked to other conditions, such as dementia, cognition, pregnancy outcomes and reproductive health [52–54]. The inclusion of only some of the health outcomes likely results in an underestimation of the total health burden.

### Strengths and limitations

This study provides a first detailed insight into the Barcelona port-sourced air pollution contribution and associated health impacts and the spatial distributions thereof, and hence is different from previous studies that addressed health impacts of shipping emissions at city or country-level, using different emission inventories and modelling techniques [19, 20]. This local spatial insight is useful to inform local policy and also pinpoint which neighbourhoods are most strongly affected and need intervention most urgently.

The modelling of port-sourced air pollution included non-ship activities, i.e., auxiliary land machinery and road traffic, which is a strength and provides a more complete picture, as these kind of port activities need consideration as well and have their own air pollution and health impact contribution, as demonstrated previously [13].

HIA studies most frequently assign exposure levels at the residential level, and therefore do not capture real-life variability in exposure according to population activity in terms of time and spatial movement [55]. This can lead to exposure and thereby health impact misclassification. Hence, it cannot be said with complete certainty if Barcelona residents of the south-eastern neighbourhoods have truly higher exposures to port-sourced air pollution (and thus suffer higher attributable health burdens), since we lack the detailed information to determine if they spend most of their day in their neighbourhood of residence. Also, HIA studies cannot consider varying susceptibilities of individuals for disease development and progression because they are based on general population estimates of expected health impacts, given established exposure-response relationships. Furthermore, our study did not account for total port emissions. Total port emissions were estimated to be quite high (i.e., 43% of total Barcelona NO_x_ emissions and 52% of total PM_10_) [28]_,_ but do not necessarily impact city air quality, thus, not having a direct effect on human health. Nevertheless, total port emissions contribute to global warming, which indirectly produces health effects for populations worldwide, including Barcelona residents, which we did not consider in this study. Finally, having used 2017 exposure, population and health data ensures reflecting some steady-state business-as-usual situation, as more recent data (e.g. 2020–2022) is probably affected by Covid-19 pandemic effects. Nevertheless, having used 2017 data, we cannot account for changes in shipping and port-sourced air pollution expected with the important 2020 IMO sulphur fuel content cap policy (i.e., reducing the sulphur fuel content to 0.1% in established SECAs and 0.5% in non-SECA waters) [43] that is expected to have an important impact on shipping and port-sourced air pollution levels [12] and has previously been estimated to reduce shipping-sourced air pollution (PM_2.5_) related mortality by 34% globally [10], by 46% for the Iberian Peninsula [20], and by 7% for Barcelona [19]. A repetition of this study, once Covid-19 pandemic effects have balanced out, and the business-as-usual situation for Barcelona’s shipping demands and port activities is more established and data is available, will provide added value.

## Conclusions

In conclusion, a considerable health burden was estimated to be attributable to port-sourced NO_2_, PM_10_ and PM_2.5_ concentrations in 2017 for Barcelona. The port-sourced air pollution contribution and associated health burden was highest for Barcelona neighbourhoods in the south-east and gradually decreased towards the neighbourhoods in the north-west. Cities need local, spatial insight into environmental risks, their sources, and associated health burdens, as they are more agile to act than national governments in developing targeted intervention strategies and policies. Shipping and port-sourced air pollution is an understudied pollution source that needs more research as well as policy attention to protect the environment and human health.

## Supporting information

S1 File Supplementary material containing S1-S4 Tables and S1 Fig. (DOCX)

## Abbreviations

BR: Barcelona Regional
CERC: Cambridge Environmental Research Consultants
CI: Confidence interval
CRA: Comparative risk assessment
CVD: Cardiovascular disease
ECA: Emission control area
EEDI: Energy Efficiency Design Index
ERF: Exposure response function
EU: European Union
GHG: Greenhouse gases
HIA: Health impact assessment
IMO: International Maritime Organisation
LNG: Liquified natural gas
MARPOL: International Convention for the Prevention of Pollution from Ships
NECA: Nitrogen emission control area
NO_x_: Nitrogen oxides
NO_2_: Nitrogen dioxide
PI: Prediction interval
PM: Particulate matter
PM_2.5_: Particulate matter ≤2.5 μm
PM_10_: Particulate matter ≤10 μm
PMQA: Pla de millora de la qualitat de l’aire
SECA: Sulphur emission control area
SEMMP: Ship Energy Efficiency Management Plan
SO_x_: Sulphur oxides
TRAP: Traffic-related air pollution
UN: United Nations
