# Supplementary material for: Health impact assessment of port-sourced air pollution in Barcelona
Source: PLoS One. 2024 Aug 30;19(8):e0305236. doi: 10.1371/journal.pone.0305236 (PMC11364232; doi:10.1371/journal.pone.0305236)
Supplement: S1 File — (DOCX) [file pone.0305236.s001.docx]

**Supplementary material**

**Supplementary tables**

**S1 Table. Barcelona adult population by age and sex (2017)**

| **Age range** | **Males** | **Females** | **Total** |
| --- | --- | --- | --- |
| ≥ 20 years | 627,286 | 722,284 | 1,349,570 |
| ≥ 35 years | 476,189 | 565,477 | 1,041,666 |
| ≥ 65 years | 139,201 | 209,546 | 348,747 |

**S2 Table. Exposure-response functions (ERFs) for NO_2_ and selected health outcomes**

| **Health outcome** | **Risk estimate** | **Exposure** | **Study design** | **Reference** |
| --- | --- | --- | --- | --- |
| All-cause mortality | HR=1.02  (PI 0.99-1.06) | 10 µg/m^3^ | Meta-analysis | (1) |
| CVD | RR=1.01  (95% CI 1.01-1.02) | 10 µg/m^3^ | Meta-analysis | (2) |
| Hypertension | OR=1.03  (95% CI 1.01–1.06) | 10 µg/m^3^ | Meta-analysis | (3) |
| Type 2 diabetes | RR=1.08  (95% CI 1.00-1.17) | 10 µg/m^3^ | Meta-analysis | (4) |
| Stroke | RR=1.04  (95% CI 0.91-1.19) | 10 µg/m^3^ | Meta-analysis | (5) |

CVD=cardiovascular disease; HR=hazard rate; OR=odds ratio; PI=prediction interval; RR=relative risk; 95% CI= 95% confidence interval

**S3 Table. Exposure-response functions (ERFs) for PM_10_ and selected health outcomes**

| **Health outcome** | **Risk estimate** | **Exposure** | **Study design** | **Reference** |
| --- | --- | --- | --- | --- |
| All-cause mortality | RR=1.04  (95% CI 1.00-1.09) | 10 µg/m^3^ | Meta-analysis | (6) |
| CVD | RR=1.12  (95% CI 1.01-1.25) | 10 µg/m^3^ | Meta-analysis | (7) |
| Hypertension | OR=1.05  (95% CI 1.04–1.07) | 10 µg/m^3^ | Meta-analysis | (3) |

CVD=cardiovascular disease; OR=odds ratio; RR=relative risk; 95% CI= 95% confidence interval

**S4 Table. Exposure-response functions (ERFs) for PM_2.5_ and selected health outcomes**

| **Health outcome** | **Risk estimate** | **Exposure** | **Study design** | **Reference** |
| --- | --- | --- | --- | --- |
| All-cause mortality | RR=1.07  (95% CI 1.04-1.09) | 10 µg/m^3^ | Meta-analysis | (8) |
| CVD | RR=1.13  (95% CI 0.98-1.30) | 5 µg/m^3^ | Meta-analysis | (7) |
| Type 2 diabetes | RR=1.10  (95% CI 1.02-1.18) | 10 µg/m^3^ | Meta-analysis | (4) |
| Stroke | RR=1.19  (95% CI 0.88-1.62) | 5 µg/m^3^ | Meta-analysis | (5) |

CVD=cardiovascular disease; RR=relative risk; 95% CI= 95% confidence interval

**S5 Table. Life table Catalonia (2013-2017)**

| **Life table IDESCAT** (9) | | | | | | | **Years lived** | | | | | |
| --- | --- | --- | --- | --- | --- | --- | --- | --- | --- | --- | --- | --- |
| **Age** | **_n_P_x_** | **_n_D_x_** | **_n_M_x_** | **_n_q_x_** | **l_x_** | **_n_d_x_** | **Baseline** | **NO_2_ total** | **NO_2_ port** | **PM_10_ total** | **PM_10_ port** | **PM_2.5_ port** |
| 0 | 71,270 | 176 | 0.00247 | 0.00247 | 100,000 | 247 | 100,000 | 100,000 | 100,000 | 100,000 | 100,000 | 100,000 |
| 1 a 4 | 311,494 | 38 | 0.00012 | 0.00049 | 99,753 | 49 | 399,013 | 399,090 | 399,019 | 399,101 | 399,014 | 399,015 |
| 5 a 9 | 419,067 | 32 | 0.00008 | 0.00038 | 99,705 | 38 | 498,523 | 498,638 | 498,532 | 498,654 | 498,525 | 498,525 |
| 10 a 14 | 384,532 | 33 | 0.00009 | 0.00043 | 99,667 | 43 | 498,333 | 498,463 | 498,342 | 498,481 | 498,335 | 498,335 |
| 15 a 19 | 348,782 | 61 | 0.00017 | 0.00087 | 99,624 | 87 | 498,119 | 498,266 | 498,130 | 498,286 | 498,121 | 498,122 |
| 20 a 24 | 361,333 | 94 | 0.00026 | 0.00130 | 99,537 | 129 | 497,684 | 497,864 | 497,697 | 497,889 | 497,686 | 497,687 |
| 25 a 29 | 415,662 | 123 | 0.00030 | 0.00148 | 99,407 | 147 | 497,037 | 497,268 | 497,054 | 497,299 | 497,040 | 497,041 |
| 30 a 34 | 517,074 | 193 | 0.00037 | 0.00186 | 99,260 | 185 | 496,302 | 496,590 | 496,323 | 496,629 | 496,305 | 496,307 |
| 35 a 39 | 642,111 | 328 | 0.00051 | 0.00255 | 99,075 | 253 | 495,377 | 495,736 | 495,403 | 495,786 | 495,381 | 495,383 |
| 40 a 44 | 643,537 | 560 | 0.00087 | 0.00434 | 98,823 | 429 | 494,113 | 494,570 | 494,146 | 494,633 | 494,118 | 494,121 |
| 45 a 49 | 585,149 | 930 | 0.00159 | 0.00792 | 98,394 | 779 | 491,968 | 492,590 | 492,013 | 492,676 | 491,975 | 491,978 |
| 50 a 54 | 527,757 | 1,453 | 0.00275 | 0.01367 | 97,615 | 1,335 | 488,074 | 488,994 | 488,140 | 489,121 | 488,084 | 488,089 |
| 55 a 59 | 468,678 | 2,068 | 0.00441 | 0.02182 | 96,280 | 2,101 | 481,401 | 482,827 | 481,503 | 483,024 | 481,417 | 481,425 |
| 60 a 64 | 410,144 | 2,686 | 0.00655 | 0.03222 | 94,179 | 3,034 | 470,896 | 473,104 | 471,054 | 473,410 | 470,921 | 470,933 |
| 65 a 69 | 379,604 | 3,662 | 0.00965 | 0.04710 | 911,45 | 4,293 | 455,725 | 459,033 | 455,962 | 459,493 | 455,762 | 455,780 |
| 70 a 74 | 308,648 | 4,555 | 0.01476 | 0.07116 | 86,852 | 6,181 | 434,261 | 439,063 | 434,604 | 439,732 | 434,314 | 434,340 |
| 75 a 79 | 252,258 | 6,567 | 0.02603 | 0.12221 | 80,672 | 9,859 | 403,358 | 410,175 | 403,842 | 411,127 | 403,432 | 403,469 |
| 80 a 84 | 233,586 | 11,020 | 0.04718 | 0.21100 | 70,813 | 14,942 | 354,063 | 363,736 | 354,748 | 365,094 | 354,168 | 354,220 |
| 85 a 89 | 153,833 | 13,460 | 0.08750 | 0.35897 | 55,871 | 20,056 | 279,355 | 292,386 | 280,270 | 294,235 | 279,495 | 279,565 |
| 90 a 94 | 70,871 | 10,575 | 0.14921 | 0.54337 | 35,815 | 19,461 | 179,076 | 194,242 | 180,124 | 196,434 | 179,237 | 179,317 |
| 95 i més | 15,934 | 4,676 | 0.29346 | 1.00000 | 16,354 | 16,354 | 55,729 | 70,090 | 56,643 | 72,377 | 55,868 | 55,937 |
| Total years lived for population ≥ 20 years | | | | | | | 6,574,420 | 6,648,267 | 6,579,524 | 6,658,959 | 6,575,201 | 6,575,591 |
| Average life expectancy for population ≥ 20 years | | | | | | | 66.05 | 66.79 | 66.10 | 66.90 | 66.06 | 66.06 |

_n_P_x_=number of persons in population; _n_D_x_=number of observed deaths; _n_M_x_=age-specific death rates; _n_q_x_=probability of dying; l_x_=number of persons alive at beginning of interval_; n_d_x_=number of persons dying during age interval

To calculate average life expectancy for the population ≥ 20 years, the total number of years lived between age 20 and ≥ 95 was calculated by multiplying the number of survivors in each of the five-year intervals (represented by l_x_) by 5. The result is the number of years lived between ages 20 and ≥ 95 for the Barcelona population. Average life expectancy (i.e. baseline) for the population ≥ 20 years is defined as the total number of years lived (i.e. 6,574,420) divided by the total number of subjects alive at age 20 (99,537 out of each 100,000).

The life table analysis represents the expected average change in life expectancy one could expect if reported total and port-sourced air pollution concentrations were set equal to zero in comparison to baseline (i.e. avoided loss in life expectancy).

**Supplementary figure**


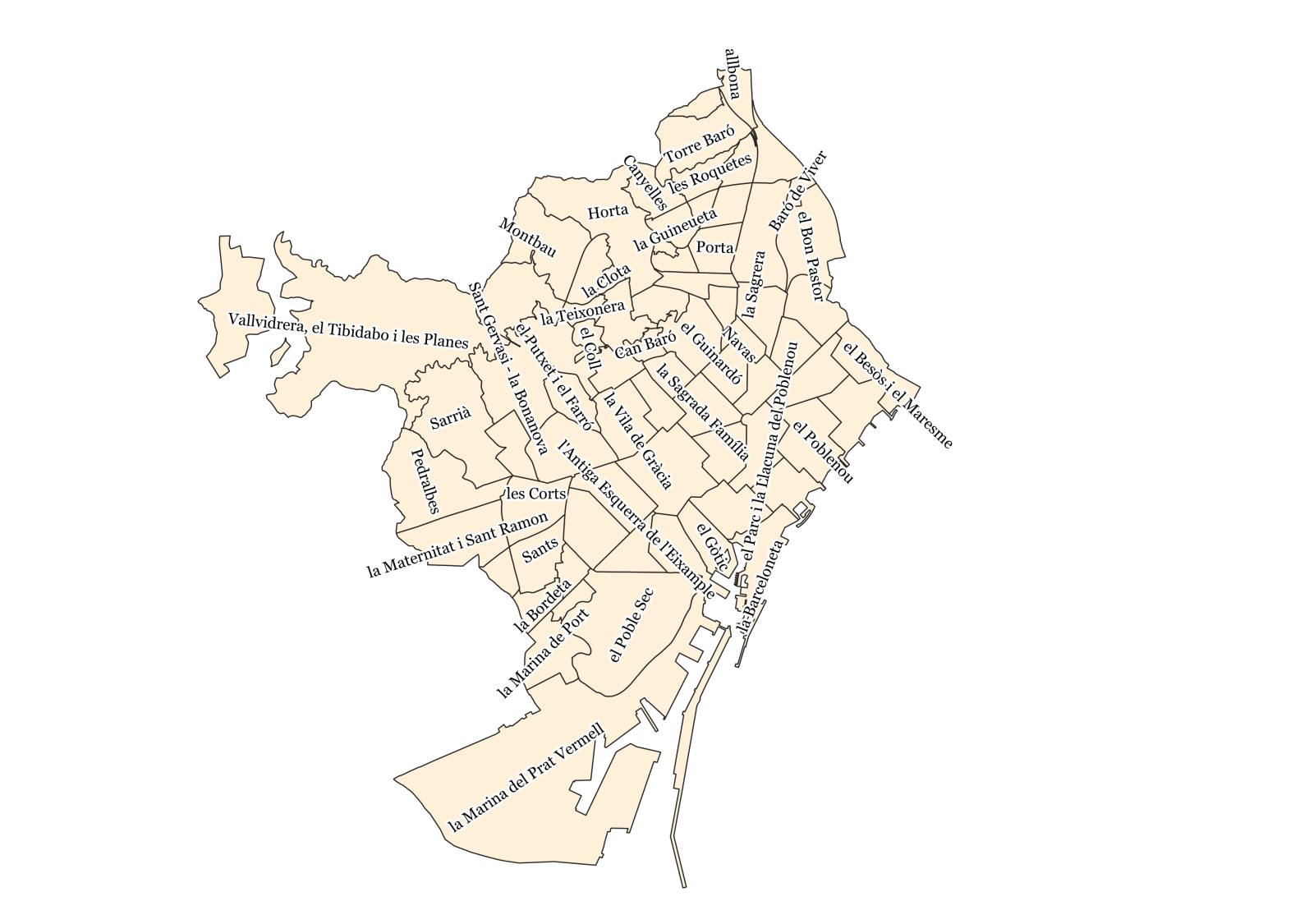
**S1 Fig. Barcelona’s 73 administrative neighbourhoods**

**References**

1. Atkinson RW, Butland BK, Anderson HR, Maynard RL. Long-term concentrations of nitrogen dioxide and mortality. Epidemiology. 2018;29(4):460–72.

2. Mustafic H, Jabre P, Caussin C, Murad M h, Escolano S, Tafflet M, et al. Main air pollutants and myocardial infarction: a systematic review and meta-analysis. JAMA. 2012;307(7):713–21.

3. Cai Y, Zhang B, Ke W, Feng B, Lin H, Xiao J, et al. Associations of Short-Term and Long-Term Exposure to Ambient Air Pollutants With Hypertension: A Systematic Review and Meta-Analysis. Hypertension. 2016;68(1):62–70.

4. Eze IC, Hemkens LG, Bucher HC, Hoffmann B, Schindler C, Kunzli N, et al. Association between ambient air pollution and diabetes mellitus in Europe and North America: Systematic review and meta-analysis. Environ Health Perspect. 2015;123(5):381–9.

5. Stafoggia M, Cesaroni G, Peters A, Andersen ZJ, Badaloni C, Beelen R, et al. Long-Term Exposure to Ambient Air Pollution and Incidence of Cerebrovascular Events: Results from 11 European Cohorts within the ESCAPE Project. Environ Health Perspect. 2014;122(9):919–25.

6. Beelen R, Raaschou-Nielsen O, Stafoggia M, Andersen ZJ, Weinmayr G, Hoffmann B, et al. Effects of long-term exposure to air pollution on natural-cause mortality: An analysis of 22 European cohorts within the multicentre ESCAPE project. Lancet. 2014;383(9919):785–95.

7. Cesaroni G, Forastiere F, Stafoggia M, Andersen ZJ, Badaloni C, Beelen R, et al. Long term exposure to ambient air pollution and incidence of acute coronary events: prospective cohort study and meta-analysis in 11 European cohorts from the ESCAPE Project. BMJ. 2014;348(f412):1–16.

8. WHO. WHO Expert Meeting: Methods and tools for assessing the health risks of air pollution at local, national and international level [Internet]. Copenhagen; 2014 [cited 2015 Aug 6]. Available from: http://www.euro.who.int/en/health-topics/environment-and-health/air-quality/publications

9. IDESCAT. Taula de vida abreujada. Tots dos sexes. Barcelona 2013-2017 [Internet]. 2019. Available from: http://www.idescat.cat/pub/?id=iev&n=8641&geo=prov:08&lang=es

10. Barcelona City Council. Population characteristics. Official figures of population. 2017. [Internet]. 2017 [cited 2018 Jul 17]. Available from: http://www.bcn.cat/estadistica/angles/dades/tpob/pad/ine/a2017/edat/edatq11.htm
